# Impact of a web-based treatment decision aid for early-stage prostate cancer on shared decision-making and health outcomes: study protocol for a randomized controlled trial

**DOI:** 10.1186/s13063-015-0750-x

**Published:** 2015-05-27

**Authors:** Maarten Cuypers, Romy E. D. Lamers, Paul J. M. Kil, Lonneke V. van de Poll-Franse, Marieke de Vries

**Affiliations:** Department of Social Psychology, Tilburg University, Warandelaan 2, 5037 AB Tilburg, The Netherlands; Department of Urology, St. Elisabeth Hospital, Hilvarenbeekseweg 60, 5022 GC Tilburg, The Netherlands; Department of Medical Psychology and Clinical Psychology, CoRPS - Center of Research on Psychology in Somatic Diseases, Tilburg University, Warandelaan 2, 5037 AB Tilburg, The Netherlands; Comprehensive Cancer Centre Netherlands South, Zernikestraat 29, 5612 HZ Eindhoven, The Netherlands; Department of Social Psychology, Tilburg Institute for Behavioral Economics Research (TIBER), Tilburg University, Warandelaan 2, 5037 AB Tilburg, The Netherlands

**Keywords:** Prostate cancer, decision aid, shared decision-making, implementation, values clarification methods, decisional conflict, decisional regret, treatment choice, information provision

## Abstract

**Background:**

At an early stage, prostate cancer patients are often eligible for more than one treatment option, or may choose to defer curative treatment. Without a pre-existing superior option, a patient has to weigh his personal preferences against the risks and benefits of each alternative to select the most appropriate treatment. Given this context, in prostate cancer treatment decision-making, it is particularly suitable to follow the principles of shared decision-making (SDM), especially with the support of specific instruments like decision aids (DAs). Although several alternatives are available, present tools are not sufficiently compatible with routine clinical practice. To overcome existing barriers and to stimulate structural implementation of DAs and SDM in clinical practice, a web-based prostate cancer treatment DA was developed to fit clinical workflow. Following the structure of an existing DA, Dutch content was developed, and values clarification methods (VCMs) were added. The aim of this study is to investigate the effect of this DA on (shared) treatment choice and patient-reported outcomes.

**Methods/design:**

Nineteen Dutch hospitals are included in a pragmatic, cluster randomized controlled trial, with an intervention and a control arm. In the intervention group, the DA will be offered after diagnosis, and a summary of the patients’ preferences, which were identified with the DA, can be discussed by the patient and his clinician during later consultation. Patients in the control group will receive information and decisional support as usual. Results from both groups on decisional conflict, treatment choice and the experience with involvement in the decision-making process are compared. Patients are requested to fill in questionnaires after treatment decision-making but before treatment is started, and 6 and 12 months later. This will allow the development of treatment satisfaction, decisional regret, and quality of life to be monitored. Clinicians from both groups will evaluate their practice of information provision and decisional support.

**Discussion:**

This study will describe a web-based prostate cancer treatment DA with VCMs. The effect of this DA on the decision-making process and subsequent patient reported outcomes will be evaluated.

**Trial registration:**

The Netherlands National Trial Register: NTR4554, registration date 1 May 2014.

## Background

Prostate cancer (PrCa) is the most common malignancy in men in the western world, and in The Netherlands where more than 10,000 new prostate cancer patients are diagnosed each year [1]. Incidence is still growing due to earlier detection and an ageing population [2–4]. Based on demographic developments only, the incidence of prostate cancer in The Netherlands is expected to increase by 49 % between 2011 and 2030 [5].

For the treatment of localized (low and intermediate risk) prostate cancer, the most common curative treatment options include radical prostatectomy, external beam radiotherapy (EBRT), and brachytherapy. Each curative treatment option has a specific risk profile concerning the occurrence of treatment side effects (for example, impotence, incontinence, and bowel problems). Because curative treatment may not always be necessary as initial treatment for low-risk PrCa, active surveillance can be considered a valid option for avoiding or deferring the need for curative treatment. Active surveillance has some known psychosocial barriers like anxiety and uncertainty about disease progression, which can withhold patients from choosing this option, although active surveillance is increasingly applied in clinical practice [6, 7]. Clinical practice guidelines do not provide strong treatment recommendations given a lack of convincing evidence indicating superiority of any of the available options [8]. Choosing the most suitable treatment option therefore requires a patient to evaluate the treatment procedure, risk for side-effects and the chance of success for all available options. Combined with personal preferences and characteristics, identifying the best suitable treatment option is a difficult and stressful exercise for many patients [9, 10]. Further complicating factors are clinicians’ misinterpretation of patients’ preferences, (information) needs and the patient’s preferred role in the decision-making process [11–15]. Eventually, this may result in the clinician dominating in the treatment decision-making at the expense of the patients’ preferences. It is possible that expressing a dominant clinician view may contribute to observed regional variations in the management of prostate cancer [16–20].

During the past decade, several decision aids (DAs) have been developed with a special focus on prostate cancer care. Instruments range from information booklets to tailored web-based tools. The variety in the formats used may have contributed to the finding that effects on decisional outcomes have been inconsistent across randomized trials and that no effects on choice have been found [21, 22]. Systematic reviews further emphasize that many previous studies are at high risk of selection bias due to inadequate concealment or blinding of data collectors and outcomes assessors, and that more studies are needed to determine how DAs can be implemented best in clinical practice [21, 22].

Determining the effect of a DA intervention and finding optimal implementation methods are both aims of the current trial. A web-based prostate cancer treatment DA was developed to fit with Dutch clinical workflow. Based on the structure of an existing DA developed by Feldman-Stewart and colleagues [23, 24], Dutch content was written and values clarification methods (VCMs) were added. Adaptation of the DA was based on the International Patient Decision Aid Standards (IPDAS) [25].

## Methods/design

### Objectives and hypothesis

The main objective of this study is to investigate the impact of the DA on shared decision-making and treatment choice. It is hypothesized that DA usage will improve prostate cancer knowledge and satisfaction with information provision and therefore better prepare patients for clinical encounters and the following decision-making, which will result in lower levels of decisional conflict compared to standard care. Further, it is expected that better knowledge and less decisional conflict will also result in improved treatment satisfaction, less regret and, ultimately, improved health-related quality of life (HRQoL). In terms of actual choice, we expect less variation in selected treatments in the intervention group compared to the control group.

A secondary aim is to investigate optimal implementation, as previous studies have emphasized the need to gain more insight on this matter [21]. From the patient perspective, it is expected that some subgroups will experience more benefit from DA usage than others. To identify these groups, the moderating role of age, preferred role in the decision-making process, specific health skills (for example, health numeracy and literacy) and personality on the main outcomes will be investigated. Healthcare providers in the intervention group will be asked their opinion about implementation of the DA. This will be compared with an evaluation of information provision and decisional support as provided by healthcare providers in the control group.

### Study design

The design for this study is a two-armed pragmatic, cluster randomized controlled trial (CRCT). Clustering is performed at the hospital level, meaning that all included patients from a participating hospital are in the same study group. Participating hospitals can therefore provide the same type of care to all of their patients, making a CRCT less prone to contamination bias [26]. The study will be longitudinal, including patients immediately after prostate cancer diagnosis and following them for 12 months. Patient-reported outcomes from both arms will be compared. Involved healthcare professionals will be included in a survey-study to evaluate their opinion on working with the DA. A comparison will be made with procedures of usual care from the control group.

The description of this design follows the CONSORT recommendation for reporting on trials (www.consort-statement.org) with the extensions for pragmatic [27] and cluster [28] randomized trials.

### Randomization

Nineteen Dutch hospitals have been randomized to either ‘usual care’ (arm 1) or ‘usual care + DA’ (arm 2). With this so-called prerandomization, the conventional sequence of obtaining informed consent followed by randomization is reversed [29]. This prerandomization is needed because we make clinicians aware (when introducing the DA) of the principles of shared decision-making and the characteristics of the DA. This could affect the control group if they were recruited within the same hospital.

To prevent potential imbalance between the two arms that could arise from hospital characteristics (for example, hospital size and treatment profile), two strata were included in the randomization procedure. First, if cooperation between two hospital locations leads to overlap in medical staff or patients visiting both locations, there is a risk for contamination bias if these hospital locations are not in the same cluster. In our sample, four pairs of hospitals have this overlap in hospital staff or patient visits. To maintain variability in hospital characteristics within each cluster, only two pairs were allowed to enter the same cluster. The second criterion is related to hospital specific treatment variation. In The Netherlands, hospitals that perform robot-assisted radical prostatectomy indicate a significant larger proportion of their patients for surgery compared to other hospitals [4]. At the moment of randomization, three hospitals from our sample are known for having robotic surgery facilities to treat prostate cancer patients, and only two of these hospitals were allowed to join the same cluster.

Randomization was performed by a statistician not involved in the study and blind to the identity of the hospitals, using SPSS version 19.0 (Statistical Package for Social Sciences, Chicago, IL, USA). As a first step, the four paired hospitals were randomized (block size = 2). Next, the remaining 11 hospitals were also block randomized (block size = 6, with the last position unused). A set seed was chosen that fulfilled to the criteria that only two robot facilitated hospitals were allowed into the same cluster. The generated group order was then applied to a pre-existing list of participating hospitals, which was sorted in alphabetical order. As there is an uneven number of hospitals participating in this study, the largest cluster that was formed was identified as the intervention cluster.

### Study population, inclusion criteria and exclusion criteria

The DA is developed for the initial treatment decision in early-stage prostate cancer. In order to be eligible to participate in this study, a subject must meet the following inclusion criteria:

1. Patient is diagnosed with low or intermediate risk prostate cancer (EAU/ESTRO criteria) [30].
2. Patient is eligible for at least two of following treatment options: active surveillance, radical prostatectomy, brachytherapy, external beam radiotherapy.
3. Patient has access to a PC, laptop or tablet with an internet connection.

Exclusion criteria are:

1. A combination of PSA ≥10 and Gleason = 7 (which defines high-risk prostate cancer).
2. Cognitive impairment or being too ill at time of the study.
3. Insufficient understanding of the Dutch language to complete questionnaires and understand the DA.

### Intervention

After being diagnosed with prostate cancer, but before a treatment decision has been made, patients in the intervention arm receive access to the online DA. Healthcare providers are instructed to introduce the DA and the study at diagnosis. However, the pragmatic nature of this trial allows hospitals to integrate the introduction of the DA with their standard information provision routines if that follows later due to follow-up diagnostics or an additional consultation with (oncology) nurses. In daily practice, this means that either the urologist or the (oncology) nurse introduces the DA to the patient. To use the DA, patients receive a card from their urologist stating their relevant disease characteristics (PSA, Gleason, and eligible treatment options) and a personal username and password to gain online access to the DA. If a nurse introduces the DA and accompanying access card, the urologist should provide the requested clinical characteristics to the nurse, either by filling in the card or by leaving a note in the patients’ record.

The DA offers a stepwise guidance through the decision process. In the first step, general information about prostate cancer is provided. The second step offers the consideration between active surveillance and curative treatment (surgery or radiotherapy). Values clarification statements are presented in this step to elicit a patient’s preference based on three main differences between AS and curative treatment; acceptance of deferring treatment (*‘I am confident enough that I will be treated on time*, *if necessary’* versus ‘*I do not want to postpone treatment because I do not want to be too late*’), avoiding possibly unnecessary treatment (‘*If treatment might be unnecessary, I would rather wait*’ versus ‘*I prefer treatment, even if it might be unnecessary*’) and the acceptance of treatment side effects (*‘I find possible treatment side effects like erectile and urinary dysfunctions difficult to accept’* versus ‘*I find the possible treatment side effects acceptable’*). Each statement is related to one of the two offered treatment alternatives in this step. On a slider scale, patients can indicate for each set of statements the strength of their preference towards one of the alternatives.

Following the same structure, the next step supports the consideration between surgery and radiotherapy. For surgery, three common methods are discussed (laparoscopic, open and robot assisted). For radiotherapy this consists of brachytherapy and EBRT. Again, information provision is followed by values clarification statements. The VCMs in this step emphasize the main differences between surgery and radiation therapy (both brachy and EBRT) in terms of treatment procedure (‘*I find it important that all cancer cells are removed from my body’* versus ‘*I find it important that the cancer cells die and not grow further*’), side effects (‘*I find bowel problems worse than incontinence*’ versus ‘*I find incontinence worse than bowel problems’*), secondary treatment (*‘I am comforted by the thought that I can have radiation if surgery is unsuccessful’* versus ‘*I accept that surgery is difficult after radiation’*) and fear for surgery (*‘I am not anxious about surgery’* versus *‘I am anxious about surgery’*). If a patient already indicated a preference for active surveillance in the previous step, the program allows patients to ignore this part and continue to the last step. As a conclusion, the final step asks patients to indicate their final treatment preference and briefly explain their choice. The DA does not provide treatment advice, but helps the patient to reach an informed preference. A summary then provides an overview of all answers to the statements and the patients’ final preference. To discuss this summary with their urologist, the summary can be printed or accessed online during the next consultation.

All statements used in the VCMs were developed by a team of urologists, psychologists and engineers based on previous experience and observation of conversations where treatment decisions were discussed. The statements were evaluated during usability-testing among patients, urologists and nurses (N = 10).

### Recruitment

Patients in both arms will be recruited by their treating urologist. When meeting the inclusion criteria, the urologist will use a letter and a leaflet, in which the study is clarified, to invite eligible patients to participate. The letter and accompanying leaflet about the study will state that we would like to investigate the information provision and decision-making process in general, without explicitly mentioning that a DA is the subject of this study. This reduces potential bias from emphasizing that the DA is an addition to usual care, as the perception of any addition to usual care may evoke improved satisfaction on itself. It will also avoid a situation where patients in the control group feel that they are withheld from a potential helpful tool. Patients are not informed about the randomization at the hospital level.

For all logistics involved to distributing and processing the questionnaires, the PROFILES-application will be used. ‘Patient Reported Outcomes Following Initial treatment and Long term Evaluation of Survivorship (PROFILES)’ is a registry for the study of the physical and psychosocial impact of cancer and its treatment from a dynamic, growing population-based cohort of both short and long-term cancer survivors. PROFILES was developed in 2009 by a joint research group from Tilburg University and Comprehensive Cancer Centre South (CCCS) with a grant from the Netherlands Organization for Scientific Research (NWO) [31]. PROFILES enables data collection management, from inviting patients to participate in studies to collecting patient-reported outcomes data via web-based or mailed questionnaires, and provides a supporting helpdesk. Patients can send their informed consent form, which they receive with the information letter to PROFILES. On the informed consent form patients can indicate whether they want to receive questionnaires via email or regular mail. Approximately 1 to 2 weeks after treatment decision-making (T1) patients receive either the invitation (email) to fill in an online questionnaire or a paper version at their home address. In the case of a paper version, a stamped self-addressed envelope is provided to the patient to return the questionnaire. If patients do not fill in their questionnaires within two weeks, a reminder letter or email will be send. Patients will be assured that nonparticipation does not result in differential follow-up care or treatment. The PROFILES-application allows for managing the follow-up questionnaires, which are sent at 6 and 12 months following T1.

### Outcome measures

Table 1 shows an overview of all outcome measures and the moment of measurement.

**Table 1:** Outcome measures

Outcomes	Instrument	T1	T2	T3
Shared decision-making outcomes				
Decisional conflict	Decisional Conflict Scale	X		
Decisional regret	Decisional Regret Scale		X	X
PrCa Knowledge	PrCa decision quality instrument	X		
Satisfaction with information	SCIP-B	X		
Decision-making preparedness	Preparation for DM-scale	X		
Decision-making role	PSDM-scale	X		
Perceived doctor-patient relationship	Single item	X	X	X
Actual treatment choice and health outcomes				
Initial preference and treatment choice	Single items	X		
Treatment satisfaction	Single item		X	X
Health-related Quality of Life	EORTC QLQ-C30	X	X	X
	EORTC QLQ-PR25	X	X	X
Side-effect impact	IIQ-7 subset for emotional state		X	X
Acceptance & Control over health status	Subjective experienced health (SBG)	X	X	X

### Primary outcomes

Primary outcome is decisional conflict. The decisional conflict scale (DCS) [32, 33] evaluates the level of decisional conflict on five subscales; the feeling of being well-informed; the clarity of values; the feeling of support during the decision-making process; the feeling of uncertainty about best choice; and the effectiveness of the decision. The level of decisional conflict is measured at T1. The DSC is widely accepted and applied as main outcome measure in (PrCa) DA trials [24, 34–36].

### Secondary outcomes

Secondary outcome measures can be categorized as either shared decision-making outcomes or health outcomes. Shared decision-making outcomes consist of decisional regret [37], perceived and preferred decision-making role [38], and preparation for decision-making to assess a patient’s preparation for decision-making and dialoguing with his clinician [39]. Furthermore, a single-item question will evaluate the perceived patient-doctor relationship and the development of this relationship over time. Also, satisfaction with information provision [40] and knowledge [41] will be assessed.

Health outcomes refer to the actual treatment choice and any changes in treatment preference during the decision-making process. Treatment satisfaction will be measured with a single-item question: ‘Are you satisfied with the way your treatment was or is executed?’ To assess health-related quality of life (HRQoL) the EORTC QLQ-C30 [42] will be used. This questionnaire is developed specific to assess HRQoL in cancer patients. Much of the content of the questionnaire is appropriate for extended monitoring of health status, including scales assessing physical, role, cognitive and emotional functioning, fatigue and sleep problems, and overall health and quality of life. This core instrument is supplemented with the prostate cancer-specific HRQoL questionnaire EORTC QLQ-PR25 [43]. This 25-item questionnaire assesses urinary, bowel and sexual symptoms and functioning, and the side-effects of hormonal treatment, though hormonal treatment is not offered as initial treatment in this study’s sample. Health outcomes are further assessed by means of an evaluation of side-effect impact [44, 45] and health status acceptance and subjective control [46].

### Other measures

Table 2 shows an overview of the other measures. Decision aid users are asked to evaluate the DA by indicating for 25 statements if it applies to the responder or not. The first 11 statements are formulated negatively (for example, ‘I found the decision aid too difficult’), followed by 14 positively formulated statements (for example, ‘I found the decision aid pleasant to use’). Although literature reports positive effects from the usage of DAs in general [47], there may be subgroups that will not benefit from DA-usage. To identify these subgroups, additional measures on (health) skills and personality are included. Objective measures for health literacy and health numeracy are used, with the HRS Experimental numeracy module [48] and the STOHFLA-brief [49], respectively. The Decision Self-Efficacy Scale is used as a subjective measure of the perceived ability to make a decision [50].

**Table 2:** Other measures

Measures	Instrument	T1	T2	T3
Implementation (intervention only)				
DA-Acceptability	Self-developed	X		
(health) skills				
Self-efficacy	Decision self-efficacy scale	X		
Health numeracy	HRS Experimental numeracy module		X	
Health literacy	STOHFLA-brief		X	
Psychosocial variables				
Anxiety and depression	HADS, PC-max	X	X	X
Personality	LOT-R, BFI-10	X		
Information seeking preferences	API, NFC-short, Maximization scale	X		
Sociodemographics and other healthcare utilization		X	X	X

Comparable to health skills, the relation of personality to beneficial DA-usage will be investigated. Some studies suggest a link between personality and treatment choice [51, 52]. Following these studies, some relevant aspects of personality will be taken into account: hospital anxiety and depression (HADS-scale) [53], prostate specific anxiety (MAX-PC) [54, 55], optimism (Life Orientation Test - Revised) [56], the big five personality dimensions (Big Five Inventory-10) [57], information seeking preferences (subscale from the Autonomy preference index) [58] and maximization tendencies (Maximization scale) [59].

### Sociodemographic variables and additional healthcare utilization

Standard sociodemographics will be asked on age, marital status, occupation, and education. Also, patients will be asked to report any additional healthcare utilization (general practitioner or other medical specialist) in the past 12 months to assess whether this affects the decision-making process.

### Healthcare providers’ evaluation

Healthcare providers in the intervention arm will be asked their opinion about implementation of the DA in qualitative interviews as well as questionnaires at the end of patient inclusion (approximately after 12 months). This questionnaire will be based on the MIDI-instrument [60], which is developed for the evaluation of implementing an innovation in a healthcare setting. The questionnaire will focus on usage of the DA (for example, *‘Did you offer the DA to all eligible patients?’*) and evaluate the pros and cons of the DA with help of statements (for example, ‘*The DA is practical in use’*). Healthcare providers in the control condition will be asked to evaluate the current information provision and decision-making processes, their expectation of DA-usage and motivation for implementation.

### Sample size calculation

The sample size for this study is determined by power analysis with decisional conflict as the primary measure. To be able to detect a clinically relevant minimum effect size [61] of .50, power is set at .80 and alpha at .05. With 19 hospitals (clusters) that agreed to participate, it is needed to estimate the intra-class coefficient (ICC). The ICC assesses the proportion of variance explained by clusters. Higher ICC values decrease effective sample size and statistical power. ICC ranges from 0 to .1 are considered common in medical literature [62]. A more specific review of ICC values in (cluster) RCTs with psychosocial measures is provided by Bell and McKenzie [63], which also included a cluster RCT evaluating a group support tool for prostate cancer patients [64]. The median estimated value for 82 longitudinal ICCs from 15 included studies was 0.0007, and the range found for decisional conflict was between 0 and 0.02. Given the considerable variability in ICCs that is found in literature, ICC for the current trial is set conservative at 0.01.

The attrition rate is set at 25 % to compensate for non-response to the questionnaires. This rate is comparable to studies in similar populations and following the same methods as this study does [65, 66]. Calculations show that a design with 19 clusters of 20 patients (380 patients in total) achieves a power of .8. Taking into account a 25 % attrition rate between the first and third questionnaire, the total sample size (rounded) will be set at 475 patients. This results in a recruitment of 25 patients per hospital.

### Statistical analysis

All analyses will be conducted using SPSS version 19.0 (Statistical Package for Social Sciences, Chicago, IL, USA). A 0.05-significance level will be adopted in all statistical tests.

We will perform a descriptive statistical analysis of organizational (hospitals) and socio-demographic (patients) characteristics in order to assure the comparability of the intervention and control groups. Baseline measures and changes in outcome variables over the study period for each study arm will be presented as means (± SD).

The main outcome decisional conflict is measured at T1 and will be compared between both groups (intervention and control). Multilevel modelling will be carried to take the hierarchical structure of the data into account by specifying random effects at both hospital and patient level. The least square mean proportions will be estimated and compared to assess the effect of the DA on decisional conflict.

The secondary outcomes will also be compared between both groups using multilevel modelling. Some of the secondary measures consist of repeated measures (for example, HRQoL and decisional regret) and will be treated according the appropriate mixed-model approach; that is, repeated measures anova/ancova will be used for outcomes with two time points (decisional regret, treatment satisfaction), and a random coefficient approach will be used for outcomes with three time points (HRQoL) [67]. Observed variation in treatment choice during the trial period will be compared between groups and at level of the individual hospital. For this second comparison, each hospital’s particular historical treatment variation profile (2008 to 2012) will be obtained from the Netherlands Cancer Registry.

Potentially confounding variables (for example, personality, health skills, and age) will be explored for their impact on the primary and secondary outcomes. Missing data and drop-outs will be described.

### Ethical considerations

The research protocol was examined by the accredited regional Medical Research Ethics Committee ‘METC Brabant’, and concluded that participants are not subjected to any procedure or imposed to perform any behavior. With this conclusion the obligation to fulfill the specific requirements of the Dutch law for Medical Research involving Human Subjects (WMO) was waived (reference: NW2014-03). The science committee of the initiating hospital has approved the study protocol (reference: WB/mt/14.030). All participating patients will sign an informed consent form.

## Discussion

This study investigates the effect of an interactive, web-based, treatment decision aid for early-stage prostate cancer. It compares impact on the decision-making process and patient-reported outcomes from an intervention group with a control group. Included patients will be followed for 12 months to investigate long-term consequences from the intervention on regret, treatment satisfaction and quality of life. Randomization will take place at the hospital level, meaning that once included, all patients within in one hospital receive the same treatment. Compared to randomization on the level of the patient, this design is less prone to contamination bias. The strength of this study is the initial involvement of 19 participating hospitals. With this large number, a proper variation of local circumstances can be taken into account that might affect structural adaptation of the DA in clinical practice. The large number of participating hospitals also requires careful management by the researchers during the trial period. Motivating all involved doctors, nurses and assistants requires careful monitoring of inclusion progress per location and adaptation to specific circumstances. Another challenge is to take the treatment variation per hospital into account. If past-year treatment characteristics appear to be imbalanced between both arms, we may decide to adjust for past year treatment, based on hospital-specific treatment profiles obtained from the Netherlands Cancer Registry.

Although we are aware of the fact that individual differences between clinicians could also affect decision outcomes, there are some considerations that justify taking the institution as the unit of analysis. First, diagnosis and offered treatment plans are often the result of multi-disciplinary consideration (for example, urologists, radiotherapists, and oncologists). Secondly, specialization often leads to some clinicians seeing the majority of PrCa patients within an institution. Taking the clinician as unit of analysis could lead to too small clusters in some cases. On the other hand, clinician specialization could also lead to patients visiting multiple clinicians within a hospital before a final decision is made, making it difficult to attribute a treatment decision to a certain clinician. Third, information provision and decisional support is often provided by specialized (oncology) nurses. Typically they assist more than one clinician, which could contaminate individual clinicians’ data. Finally, regional variation in treatment practices, which is expected to be influenced by DAs as explained in previous sections, is generally reported at the hospital level. This indicates that there are influences at the hospital level driving practice variation that go beyond individual differences between clinicians within a hospital. The reported variation in selected treatments between hospitals is available for hospitals included in our study, though no data is available on individual clinician’s variability.

While carefully designed and reviewed by experts, some content of the DA can remain the subject of discussion among healthcare providers. As every urologist, radiotherapist or nurse can be seen as an expert on prostate cancer from their own perspective; all have their own preference in formulating and presenting options, facts and risks involved to prostate cancer and its treatment alternatives. The original DA is tested thoroughly and documented for the topics that should be addressed in the DA, which we have taken over [23, 24]. All adjustments that were made to adjust the DA to Dutch clinical setting are based on the IPDAS criteria [25] for DA development. All textual content is derived from Dutch and European treatment guidelines.

A potential limitation of our DA is that a device with internet connection is needed to use the DA, which could affect our sample and consequently our findings. Although we are aware that this could be a relevant issue in many regions in the world, we do not expect biased results in our trial. The World Bank has estimated internet access in The Netherlands is among the highest in the world, with 94 % of the households (2013) having internet access (www.worldbank.org). Even in older age groups (65 to 75 years) regular internet access is at 80 %, and this percentage is rapidly increasing (2013, Statistics Netherlands). Internet is routinely referred to as part of information provision in standard care. As most of our questionnaires (in both groups) are sent via email, internet access and the ability to use it is also required in both groups, assuring group comparability on this matter.

Our trial has defined decisional conflict as primary measure. As previously mentioned, the DCS is a widely accepted and applied measure in DA evaluations. However, the DCS is also subject to some discussion in the literature about its usefulness as outcome measure in DA evaluations [68]. This is mainly due to its limitation to identify a good decision as a person’s underlying sensitivity to uncertainty may not be fully represented in a high or low decisional conflict score. For example, a high score on the DCS could also represent the effort that one takes to be involved in the decision-making process and absorbing all available information and therefore becoming aware of the difficulty of the decision. Although we are aware of this potential limitation of the DCS, we believe decisional conflict represents the best available affective-cognitive outcome measure that captures the uncertainty involved to prostate cancer treatment decision-making. Uncertainty about disease progression, treatment success and side-effect impact are key elements of the decision-making process in prostate cancer care. Preliminary investigations prior to the current study taught us that decisional conflict levels are substantial; we expect that our DA will be able to reduce these levels and that this potential reduction is meaningful. For meaningful interpretation of our effects we also have additional outcome measures available that can support our findings or can indicate bias if present. Many of our secondary measures focus on the decision-making process (knowledge, satisfaction with information provision, decision-making role) rather than the outcome in terms of a ‘good’ or ‘bad’ decision, this ensures that our conclusions on the usefulness of the DA will not solely depend on interpretation of the DCS.

On a broader level, this study will augment the current paucity of information regarding the implementation of DAs in (Dutch) routine clinical practice, its impact on the treatment decision-making process and long-term effects. This will help patients and clinicians to establish optimal patient-treatment fit. As we hypothesized the effects could involve improved patient involvement and knowledge resulting in higher decision and treatment satisfaction and ultimately reduce regret and improve quality of life.

Finally, the results of this project will contribute to the increasing awareness of shared decision-making and improving patient centered care in the treatment of prostate cancer. This study can provide the scientific evidence needed to include the use of a DA in the prostate cancer treatment guidelines.

## Trial status

This trial is currently recruiting patients. The start date was August 2014. The initial patient inclusion is expected to take twelve months. Patients will be followed for one year.

## Abbreviations

CCCS: Comprehensive Cancer Centre South
CRCT: cluster randomized controlled trial
DA: decision aid
EORTC: European Organization for Research and Treatment of Cancer
HRQoL: health-related quality of life
IPDAS: International Patient Decision Aid Standards
METC: Medical Research Ethics Committee (MREC). In Dutch: Medisch Ethische Toetsings commissie
PrCa: prostate cancer
SDM: shared decision-making
VCM: values clarification method
WMO: Medical Research Involving Human Subjects Act. In Dutch: Wet medisch-wetenschappelijk onderzoek met mensen
